# Hybrid Shell Engineering of Animal Cells for Immune Protections and Regulation of Drug Delivery: Towards the Design of “Artificial Organs”

**DOI:** 10.1371/journal.pone.0020983

**Published:** 2011-06-22

**Authors:** Philippe Dandoy, Christophe F. Meunier, Carine Michiels, Bao-Lian Su

**Affiliations:** 1 Laboratory of Inorganic Materials Chemistry, Department of Chemistry, The University of Namur (FUNDP), Namur, Belgium; 2 Laboratory of Biochemistry and Cellular Biology, Department of Biology, The University of Namur (FUNDP), Namur, Belgium; 3 State Key Laboratory of Advanced Technology for Materials Synthesis and Processing, Wuhan University of Technology, Wuhan, China; Massey University, New Zealand

## Abstract

**Background:**

With the progress in medicine, the average human life expectancy is continuously increasing. At the same time, the number of patients who require full organ transplantations is augmenting. Consequently, new strategies for cell transplantation are the subject of great interest.

**Methodology/Principal Findings:**

This work reports the design, the synthesis and the characterisation of robust and biocompatible mineralised beads composed of two layers: an alginate-silica composite core and a Ca-alginate layer. The adequate choice of materials was achieved through cytotoxicity LDH release measurement and in vitro inflammatory assay (IL-8) to meet the biocompatibility requirements for medical purpose. The results obtained following this strategy provide a direct proof of the total innocuity of silica and alginate networks for human cells as underscored by the non-activation of immune defenders (THP-1 monocytes). The accessible pore size diameter of the mineralised beads synthesized was estimated between 22 and 30 nm, as required for efficient immuno-isolation without preventing the diffusion of nutrients and metabolites. The model human cells, HepG2, entrapped within these hybrid beads display a high survival rate over more than six weeks according to the measurements of intracellular enzymatic activity, respiration rate, as well as the “de novo” biosynthesis and secretion of albumin out of the beads.

**Conclusions/Significance:**

The current study shows that active mammalian cells can be protected by a silica-alginate hybrid shell-like system. The functionality of the cell strain can be maintained. Consequently, cells coated with an artificial and a biocompatible mineral shell could respond physiologically within the human body in order to deliver therapeutic agents in a controlled fashion (i.e. insulin), substituting the declining organ functions of the patient.

## Introduction

Since the original idea published in 1933 by Bisceglie [1], significant advances have been achieved to develop immuno-isolation methods for long-term transplantation of xenogenic cells or tissues. The final aim of this technology is to treat human disorders such as protein or hormone deficiencies (e.g. type 1 diabetes mellitus, factor IX of hemophilia B, Parkinson's disease or Alzheimer's disease) [2]–[5], overcoming the usual obstacles of limited supply of donor tissue or replacing the conventional drug treatments.

Generally speaking, immuno-isolation should be understood as a way to protect cells against mechanical stress and rejection from the immune system of the host body while allowing the diffusion of nutrients, oxygen and metabolites. Conceptually, they are separated by a biocompatible and semi-permeable membrane. The porosity of this cell-entrapping material is a key parameter for successful immuno-isolation. From the literature, the targeted pore size is still not clear. If a molecular weight (MW) cut-off of around 150 kDa is generally accepted [6], [7], which is the MW of the immunoglobulin G (IgG), the diameter related to this value is still debated. Nevertheless, a pore size between 5 to 20 nm is often referenced [8]–[10]. Apart from porosity issues, the robustness and the biocompatibility of the semi-permeable system are also important features for the successful transplantation of cells. The long-term efficiency of this technology is not only dependent on the final properties of the synthesised materials, but is also closely linked to the biocompatibility of the material precursors and contaminants, the synthesis pathway, as well as the ageing phase of the material. Finally, the shape and size of the system play a critical role to avoid hypoxia-induced cell death. Microencapsulation, where a small number of cells are entrapped into several microspheres, is preferred than macroencapsulation, where huge amounts of cells are entrapped in a single immuno-isolating membrane [11]. In fact, the microencapsulation approach provides a way to enhance diffusion by increasing the surface area/volume ratio.

Advances in chemical synthesis have enabled the development of various types of materials as potential candidates for entrapping fragile animal cells. During the past decade, biopolymers (e.g. alginate, alginate/Poly-L-Lysine, chitosan, collagen), organic hydrogels (e.g. polyvinylalcohol, polyethylene glycol) or inorganic oxide gels (e.g. silica) have been employed to develop a wide range of medical applications [12]–[15]. While purified alginate is nowadays the most employed and recognized biocompatible material for cell encapsulation technology [16], it suffers from a poor mechanical stability and swells with time. To overcome this problem, the alginate core has been coated by different polycations such as poly-L-lysine, poly-L-ornithine, polyethyleneimine, polyallylamine or chitosan. However, these positively charged polymers (under physiological conditions) have been reported to be toxic since they can induce inflammatory reaction and cell necrosis [17], [18]. In this context, association of alginate capsules with biocompatible silica species brings great opportunities and interests as it allows the combination of soft biocompatible component (*viz.* alginate) with a resistant and non swelling component (*viz.* silica) [19]. In fact, the mineralisation of biopolymers by silica can be used to improve their chemical and mechanical performances. In the field of animal cell encapsulation, Carturan *et al.* [20], [21] and Sakai *et al.* [22]–[24] independently reported two methods to design alginate-silica capsules. Nevertheless, until now, the silica is only deposited at the polymer surface (layer of few microns) and not within the alginate beads. Consequently, the mechanical resistance of these capsules is not optimal and could still be improved since the slow but inevitable dissolution of SiO_2_ occurs in the body fluids [25].

In this paper, novel biocompatible ceramic-like beads have been synthesised under mild conditions *via* a rapid and simple approach. The material precursors were rationally selected on the basis of cytotoxicity and immune tests. The mineralised beads have been made with two layers (alginate-silica composite/Ca-alginate) to ensure an excellent mechanical stability and a total biocompatibility over time. Besides, small spherical beads, rather than hybrid matrix, have been targeted to promote the diffusion of nutrients and metabolites. The properties of this hybrid material are investigated to explore the opportunity to develop an “artificial organ”. For instance, the entrapment of β-cells (or pancreatic islets) within such kind of materials could be used for the treatment of *Diabetes mellitus* thanks to the *in vivo* production of insulin required by the organism to maintain glucose homeostasis. In the present study, human hepatocellular carcinoma cell line (HepG2) was chosen as a cell model because pancreatic β-cells cannot be easily cultivated [26], [27]. These HepG2 cells have similar size and morphology compared to those of β-cells. Additionally HepG2 cells continuously secrete albumin and could thus be used to mimic the diffusion of insulin out of the newly formed material.

## Results and Discussion

### Design of a robust and biocompatible host material

The immobilisation of animal cells in a robust and biocompatible material is the challenge for the successful development of cell therapy. Among the vast number of materials, porous silica is promising. This transparent inorganic material is essentially chemically inert, thermally stable and mechanically strong. Even though silica is generally considered as a biocompatible material and used in medical applications, several reports suggest that it is a strong macrophage-attracting substance [28]. In fact, the cytocompatibility of the final material is closely connected to its synthesis pathway. For instance, the shape and size of silica particles formed during the encapsulation procedure can affect the cell integrity and hence trigger inflammation. Besides, it is well recognised that the interaction of silica nanoparticles with animal cells can induce oxidative stresses, cell membrane damages and possibly cell apoptosis/necrosis [29].

Generally, silica gels are obtained under mild conditions via the hydrolysis and condensation of alkoxysilanes (e.g. TMOS, TEOS). A major drawback of this pathway is the release of cytotoxic by-products (e.g. methanol, ethanol). It is well-known that short chain alcohol can affect biological membranes [30] or even destroy them. Therefore, alternative protocols have been reported such as an intermediate distillation step of the silica sol to remove most of alcohols prior to the introduction of the living cells [31]. Another strategy is based on the utilisation of organically modified precursors that liberate non- or less-toxic polyols (e.g. ethylene glycol, glycerol) [32]–[35]. Besides, Livage *et al.* reported the synthesis of porous silica structures by an acidification of diluted sodium silicate solutions [36]. This method, called aqueous route, has been used to efficiently entrap heterotrophic or photoautotrophic bacteria [37], [38]. Nevertheless, a negative aspect of this protocol is the excess of sodium ions that can disturb the cellular osmotic balance. In fact, this pathway was reported to be unsuitable to entrap fragile biological structures such as photosynthetically active organelles [39] and plant cells [40], [41].

**Figure 1:**
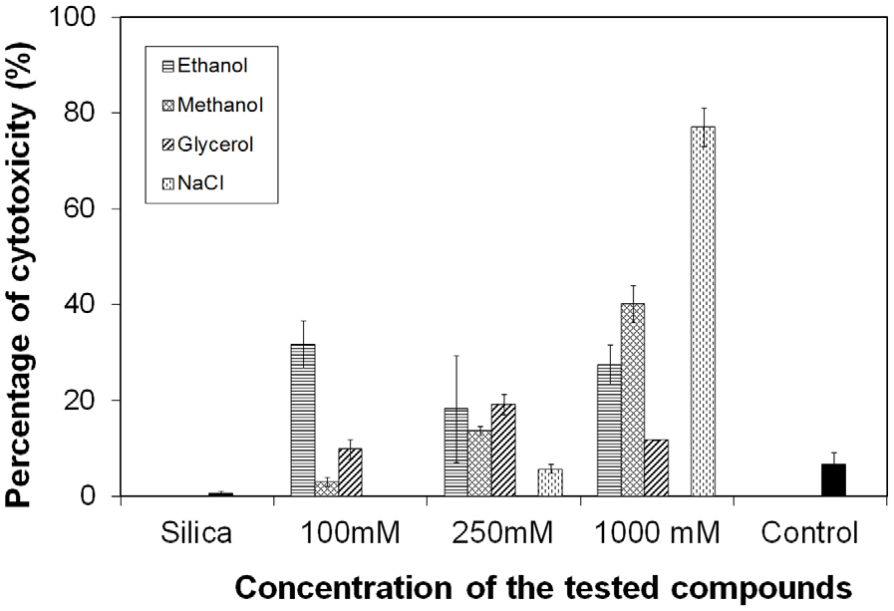
Concentration-dependent effects of different sol-gel by-products on HepG2 cells after 5 hours of incubation. The membrane damaging effect of chemicals was quantified by measuring the release of the cytosolic enzyme LDH. Results are presented in percentage of cytotoxicity as means ± 1 S.D. (n = 3).

Before performing HepG2 cell encapsulation, a cytotoxicity test was thus firstly accomplished to estimate the lethal dose of the by-products (upper limit concentration) that are formed during the different preparation methods described above. Figure 1 presents the results for the cytotoxicity test. The control is the reference; it represents the natural death of cells occurring during the experiment. For a value higher than 6.6(±2.4) %, the concentration of the tested compounds is considered to be toxic. As shown in Figure 1, ethanol was toxic at concentration between 100 mM and 1000 mM. On the contrary, the effects of methanol strongly depended on its concentration. At low concentration (below 250 mM), methanol can be considered as a non-toxic agent. However, the percentage of dead cells increased up to 40% when they were exposed to 1000 mM of methanol. Polyols such as glycerol were also significantly cytotoxic at every concentration tested, but the percentage of cells dead was lower at high concentration compared to the other aliphatic mono-alcohols. That is why it is often used as a cryoprotectant for the storage of cultured cells (at −196°C, its toxicity falls dramatically). Finally, the toxicity of sodium chloride was also measured to evaluate the relevance of gels obtained *via* an aqueous route. The data highlight that NaCl is not detrimental for the cells below a concentration of 500 mM. These results suggest that HepG2 could be entrapped within silica gel formed *via* the aqueous route (Na_2_SiO_3_ as silica precursor). This hypothesis was strengthened by the complete innocuity of aggregated silica nanoparticles (Figure 1). Nevertheless, in conventional encapsulation procedures, the introduction of silica precursors within the buffered culture medium exposes the cells to unnatural and brutal variations of their environment during the host-material synthesis. Therefore, the chemical conditions of sol-gel synthesis could be considered as harmful for human cells such as HepG2 cells. Recently, Bilmes *et al.* demonstrated that the pre-encapsulation of cells within an alginate capsule is beneficial to protect yeasts from silica precursors (e.g. sodium silicate). They showed that low concentrations of sodium alginate did not provide efficient protection to immobilised cells and too high concentrations generated ionic stress. But for alginate concentrations between 1% and 2%, the stress levels of cells were significantly lower that observed in cells directly exposed to silica precursors [42]. Based on these results, a three-step approach has been used here to encapsulate living animal cells within biocompatible hybrid alginate-silica beads.

**Figure 2:**
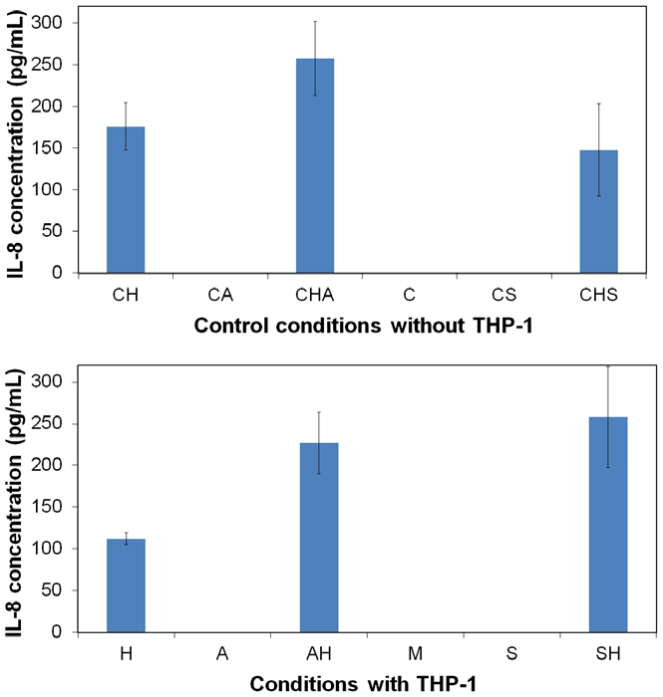
In vitro determination of a proinflammatory response due to alginate or silica around cells. Proinflammatory response is highlighted by IL-8, which is secreted by monocytes THP-1 (immune defenders) when they are activated. The control (a) are made without THP-1 to underline the basal concentration of IL-8 produced by HepG2 cells. Results are presented as means ± 1 S.D. (n = 3).

In order to confirm the biocompatibility of the building blocks used to synthesise the ceramic-like beads, an *in vitro* test for the detection of immune response was secondly performed. The objective was to determine if silica nanopowders or sodium alginate can activate immune defenders (THP-1 monocytes) to start a “rejection” mechanism. Secretion of inflammatory cytokine (IL-8) was thus assayed as a measure of macrophage activation. Before the experiment, sodium alginate (anionic polysaccharide composed of guluronic and mannuronic units) was purified to improve its biocompatibility since all the contaminants (e.g. endotoxins, proteins, polyphenols) sequestered in this crude powder are known to be toxic for animal cells [43]. Figure 2 shows the level of IL-8 secreted by THP-1 monocytes in contact with alginate or silica materials (Table 1). Without any cell (C, CA and CS), IL-8 was not detected (Figure 2a). However, it has to be mentioned that HepG2 cells constitutively secrete IL-8 (CH in Figure 2a). The presence of the alginate polymers or silica particles did not affect them as shown by steady state IL-8 concentration (CHA, CHS in Figure 2a). When THP-1 monocytes were put in contact with the materials (A, AH, S, SH in Figure 2b), the IL-8 concentration detected in the supernatants was not higher compared to CA, CHA, CS or CHS samples. These observations confirm the total biocompatibility and the inertness of purified alginate and of silica material.

**Table 1:** Conditions tested to perform the *in vitro* immune response test. Determination of IL-8 released in the supernatant, synonym of a proinflammatory response.

Conditions				
Without THP-1 (Fig. 2a)		With THP-1 (Fig. 2b)		
**CH**	Control HepG2	**H**	HepG2	THP-1
**CA**	Control Alginate	**A**	Alginate	THP-1
**CHA**	Control Alg+HepG2	**AH**	Alg+HepG2	THP-1
**C**	Control RPMI Medium	**M**		THP-1
**CS**	Control Silica	**S**	Silica	THP-1
**CHS**	Control Silica+HepG2	**SH**	Silica+HepG2	THP-1

The next section described the characterisation of robust and biocompatible mineralised beads composed of two layers: an alginate-silica composite core and a Ca-alginate layer. This bead was mineralised with silica to strengthen the polysaccharide beads. Since *in vivo* biocompatibility data have only been described in the literature for alginate, an outer layer of alginate was deposited at the surface to mask the possible unwanted interactions [44]. The performance of these novel beads was compared to conventional alginate capsules in term of long-term stability and cell viability.

### Material characterizations

**Figure 3:**
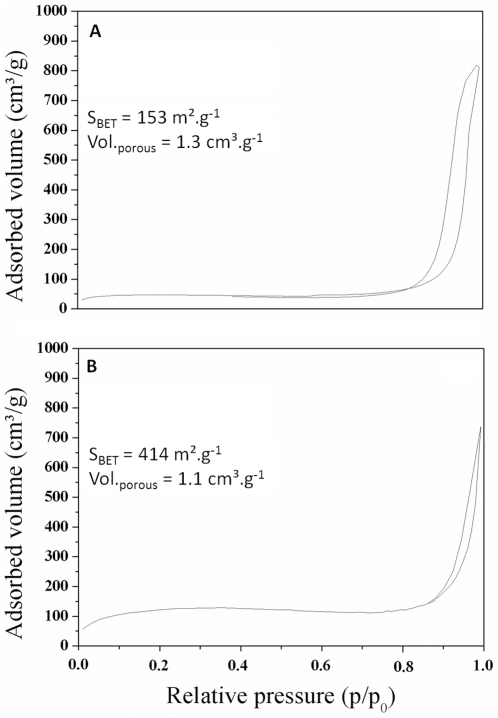
Nitrogen adsorption-desorption isotherms. Isotherms obtained by analysis of purified alginate capsules (a) and mineralised beads (b).

**Figure 4:**
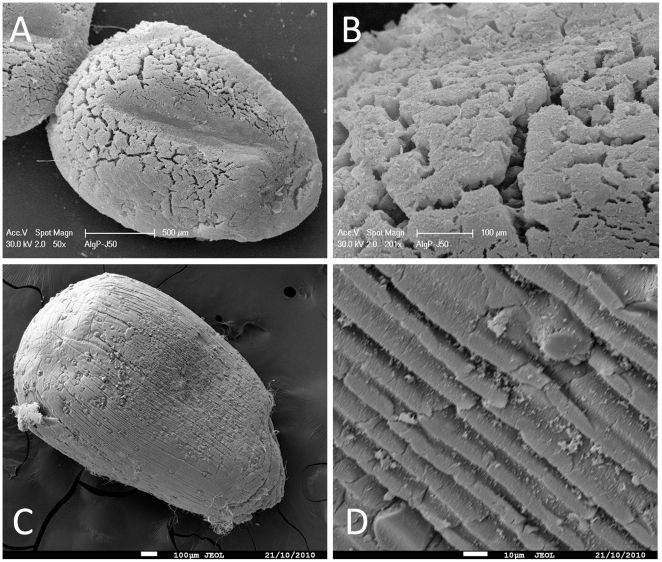
Scanning electron microscopy images of (a, b) purified alginate capsules and (c, d) mineralised beads. The materials were kept during 50 days in the culture medium and supercritically dried with carbon dioxide.

**Figure 5:**
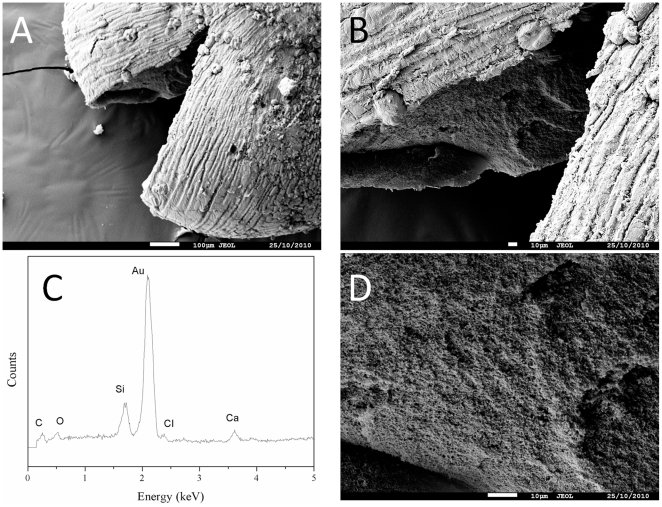
SEM micrographs (a, b, c) and EDX spectrum (d) of a cross section of a mineralised bead. The bead was conserved in vitro in the biological medium during one month.

**Figure 6:**
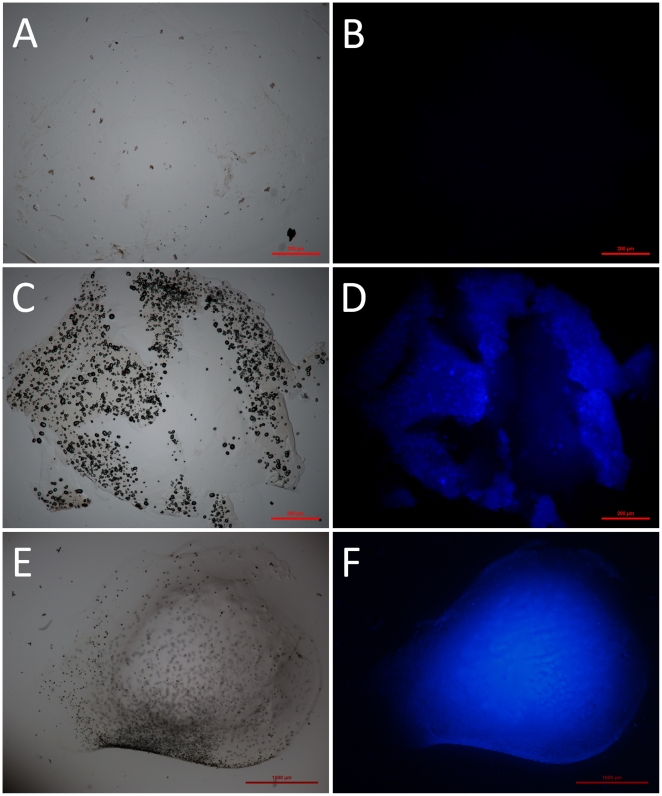
Microscopy images of an alginate capsule and a mineralized bead showing the silification. Brightfield (a, c, e) and fluorescent microscopy images (b, d, f) of an alginate capsule (a–b) and a mineralised bead (c–f) in the presence of PDMPO (silica tracer). Micrographs (a–d) and (e–f) correspond to representative cross sections of the materials and to a whole bead, respectively.

Alginate capsules and silica mineralised beads were studied to determine their properties, especially regarding their porosity which is a key parameter that determines the diffusion of chemicals including nutrients required for cell survival. After being supercritically dried with liquid carbon dioxide to preserve the porous network by avoiding the collapse of pores through capillary action, the obtained aerogels were characterised by nitrogen adsorption-desorption measurements and scanning electron microscopy (SEM). In all cases, analysis of the textural properties of materials shows a type II isotherm according to the IUPAC classification systems. The surface area of mineralised beads was very high (414 m^2^.g^−1^) compared to alginate capsules (Figure 3). This increase in surface area may suggest an efficient mineralisation of the polymeric beads and a great improvement of their mechanical properties. As a matter of fact, SEM micrographs emphasize that hybrid alginate-silica beads are more robust compared to alginate capsules. After 50 days of incubation, the alginate capsules swelled and became very fragile. Typically, large cracks (50 µm in some parts) appeared at the surface of the polymeric capsules (Figure 4a and b) while the mineralised beads presented intact surface morphology (Figure 4c and d). Besides, the core of the mineralised beads was characterised by a dense porous network that strengthens the overall mechanical properties of the material (Figure 5a, b and c). EDX analysis suggests that the three-dimensional structure of the bead core was formed via the deposition of silica (Figure 5d). In order to confirm this hypothesis, a silica tracer was used. PDMPO is an excellent fluorescent probe for imaging newly deposited silica [45]. For that purpose, hydrated alginate-silica beads and alginate capsules (50 days old) were incubated with this probe and observed with an optical microscope under an UV-excitation light. As shown by Figure 6a and b, no trace of silica species was observed within optically transparent alginate capsule. Conversely, the whole mineralised beads (Figure 6e) as well as every cross-section of the material (Figure 6c) displayed an intense blue fluorescence (Figure 6d and f). These observations indicate that silica was homogeneously distributed throughout the beads. Although, silica and alginate were both negatively charged at physiological pH, silica species can diffuse within the freshly formed alginate capsules. Since buffered culture medium present in the core part of the beads maintains the pH near 7, its favours the catalysis of silica condensation. It is assumed that the presence of weak hydrogen bonding interactions between alginate and the silanol groups promotes the formation of uniform hybrid alginate-silica composites [46], [47].

**Figure 7:**
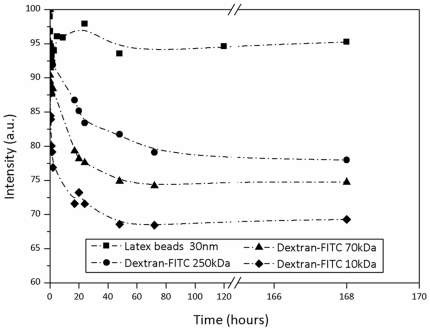
Study of the diffusion of fluorescent probes through 7 days old mineralised beads. 100% corresponds to the fluorescence intensity of the solution before the addition of the beads. The MWCO is estimated thanks to the hydrodynamic radius of biopolymer: 2, 6 and 11 nm for the dextran-FITC, and with latex beads carboxylated-modified characterized by a diameter of 30 nm.

The immuno-isolation efficiency was investigated by studying the entrance of fluorescent molecules of known diameters within 7 days old mineralised beads. Thanks to fluorescein isothiocyanate-dextran molecules with various molecular weights (10, 70 and 250 kDa), it was possible to determine whether these molecules can enter or not into the beads, the same experiment was performed with latex beads of 30 nm to test higher diameter. According to the equation of Stokes [48], the three dextran-FITC of 10 kDa, 70 kDa and 250 kDa have a diameter of 4, 12 and 22 nm respectively, where latex beads have a diameter of 30 nm. These products were chosen because they do not present significant adsorption on silica and alginate. The Figure 7 shows the results obtained over 7 days. In the case of small molecules, the bead permeability was large enough to allow a rapid diffusion of the Dextran-FITC 10 kDa within the hybrid material. After 3 days, the diffusion equilibrium was reached. The tendency was the same for the polymers characterized by a molecular weight of 70 kDa and 250 kDa. Nevertheless, these bigger molecules diffused less rapidly as proved by the gentler slope (Figure 7). Larger diameter probes, latex beads carboxylate-modified of 30 nm, where then used. In this case, there was no decrease of the fluorescent supernatant. All the latex beads stayed outside the mineralised beads (experiments were also performed with latex beads of 500 nm and 1000 nm, with the same observation – data not shown). Consequently, we assume that the pore size diameter of our materials is between 22 and 30 nm, as required for immuno-isolation.

### Metabolic activity and cell viability

**Figure 8:**
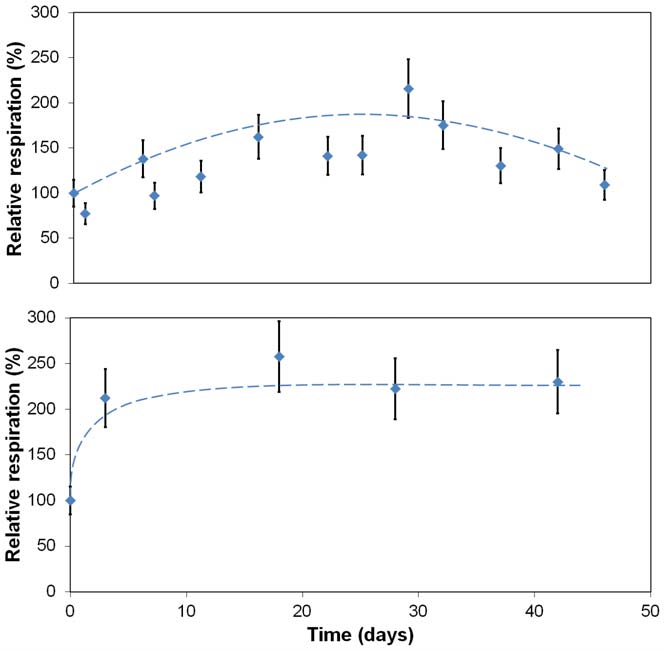
Comparison of oxygen consumption at HepG2 cells encapsulated within (a) alginate capsule or (b) mineralised bead. 100% corresponds to the oxygen consumption of cells entrapped within one bead (1.8 10^2^ µmole of O_2_ hour^−1^ bead^−1^). Results are presented as means ± 1 S.D. (n = 3).

Several characterisation methods were used to monitor the survival of cells entrapped within alginate capsules or hybrid beads. The physiological functions of HepG2 cells were firstly determined by measuring their respiration activity. The consumption rate of oxygen reflects directly the number of active cells. Results are shown in Figure 8. In purified alginate capsules, the oxygen consumption slightly increased over one month (Figure 8a). This observation indicates that the survival rate of entrapped cells was high. Nonetheless, the respiratory activity decreased after 30 days post-encapsulation. In fact, these results can be explained by the swelling behaviour and brittleness of alginate capsules. As highlighted in Figure 4b, the porosity of the alginate crust can reach 50 µm. Compared to the cell size (8 µm), these holes are large enough to allow cells to go out of the material. Consequently, the cellular density confined within the capsules analysed by oxymetry decreased as function of time. In the case of cells entrapped within mineralised beads, oxygen consumption sharply increased during the early stages following the immobilisation step (∼220%) probably due to cell proliferation. As already highlighted by Figure 4d and Figure 7, Figure 8b shows that HepG2 cells cannot leave the mineralised beads thanks to the adequate porosity of these beads. Then, the oxygen consumption remained constant during more than 42 days. This promising result also suggests that the encapsulation and the mineralisation processes are totally benign for sensitive mammalian cells.

**Figure 9:**
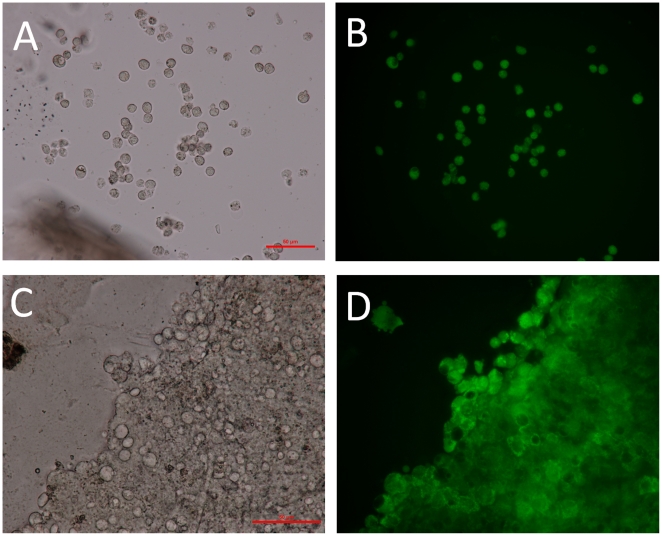
Microscopy images highlighting the survival of cells 6 weeks after immobilisation. Brightfield (a, c) and fluorescent microscopy images (b, d) of HepG2 cells three-dimensionally entrapped within a mineralised bead and labelled with fluorescein diacetate (viability probe). The micrographs were taken 1 day (a, b) and 42 days (c, d) post-encapsulation.

In order to confirm these data, cell viability was assessed by fluorescence microscopy after staining entrapped HepG2 cells with a commercially available fluorophore (FDA). If cells are still alive, they exhibit a green fluorescence due to the presence of fluorescein produced via the hydrolysis of FDA by intracellular esterase. Figure 9 shows representative micrographs of the hybrid beads taken in brightfield and fluorescent modes. One hour after encapsulation, 90% of cells, homogenously dispersed within the beads, were alive in the matrix as shown in Figure 9a and b. Forty-two days later, cells were still alive (Figure 9c and d). They were spread in a carpet-like fashion within the beads. Owing to the rigidity of hybrid alginate-silica beads, cells cannot divide and grow as they would without any constraints in culture flask. HepG2 could be able to divide one or two times as suggested by the oxygen consumption graph (Figure 9b) and by the optical micrograph (Figure 9c). However, the growth of the daughter cells would be greatly restricted by the cages that are created around them during the silica polymerisation. Indeed, the oxygen consumption rate of each bead did not gradually increase as a function of time. This is very promising results. In fact, the preservation of the mitotic activity of cells is not the first goal to target some kinds of medical applications. For instance, the synthesis of a “bioartificial pancreas” is based on the encapsulation of non-dividing cells, namely the beta-cells.

**Figure 10:**
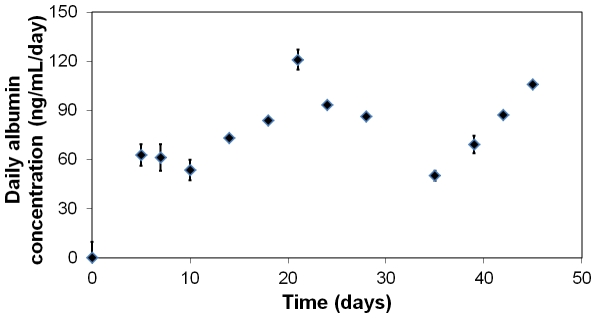
Amount of human albumin secreted by HepG2 cells in the supernatant of the mineralised beads. Concentrations showed the daily albumin released outside of the hybrid alginate-silica material as means ± 1 S.D. (n = 3).

Finally, the efficiency of the immobilisation method was validated by the human albumin produced and released in the supernatant by the entrapped HepG2 cells. Twice a week, supernatants were retrieved and replaced by fresh medium. The Figure 10 shows albumin concentration daily secreted into the supernatant. These data show that large molecules (like proteins) can diffuse through the mineralised beads, while cells remain confined within the material during a long period of time.

### Conclusions

HepG2 cells, selected as a cell model for secreting cells, were encapsulated into hybrid alginate-silica beads. This work demonstrates that whole alginate capsules can be completely and homogenously mineralised by silica. Moreover, the deposition of aqueous silica species throughout the capsules greatly improves their long-term mechanical performance. The material precursors (sodium alginate and sodium silicate) used to construct the beads are fully biocompatible as indicated by the LDH assay and by the *in vitro* immune test (IL-8). After the encapsulation step, more than 90% of cells were kept alive.

The biological activity of entrapped cells was perfectly preserved during more than 45 days as shown by the respiration activity and the secretion of albumin. Direct observations of mineralised bead by fluorescent microscopy suggest that cells were able to divide once post-immobilisation. Besides, the hybrid beads are porous, allow the diffusion of nutrients and metabolites through it and meet the requirements for successful immuno-isolation with a pore size diameter beneath 30 nm. Such highly biocompatible hybrid systems with long-term survival could contribute towards the creation of an “artificial organ” to treat human disorders. Further works are currently being carried out on *in vivo* studies to study the efficiency of the present hybrid material on animals. In the future, such kind of smart materials could be used to develop a novel organ substitution technology. Particularly, the synthesis method developed in this work could be used to entrap beta-cells in order to design a new treatment for the *Diabetes mellitus*.

## Materials and Methods

### Chemical Materials

Ethanol (99.5%, anhydrous), methanol (99.8%), D-sorbitol (99.5%), sodium chloride (99.5%), calcium chloride (dihydrate, 99%), EGTA (99%), sodium alginate powder (sodium salt from brown algae), silica nanopowder (99.5%, 5–15 nm), fluorescein isothiocyanate Dextran (Dextran-FITC) and latex beads carboxylate-modified (L5155) were provided by Sigma-Aldrich; and glycerol (p.a.), sodium silicate (assay 25.5–28.5% SiO_2_) by Merck. The Dubelcco's Modified Eagle Medium (DMEM), the Roswell Park Memorial Institute Medium (RPMI) and the foetal bovine serum were purchased from Invitrogen (Carsbald, USA). Before used, sodium alginate powder was purified according to the method described by de Vos *et al.* [43]. Briefly, this anionic polysaccharide was dissolved in an aqueous solution of EGTA 1 mM. The insoluble part of the crude commercial mixture was removed through successive filtrations (from 5 to 0.45 µm). The alginate fraction was then precipitated via the addition of HCl 2 M at 4°C. The resulting gel was successively washed with HCl 0.01 M, chloroform and butanol. A clear solution was then recovered by raising the pH to 7.0 (NaOH 0.5 M). Finally the purified alginate was precipitated via the addition of absolute ethanol and freeze-dried.

### Biological Materials

HepG2 cells (Hepatocellular Liver Carcinoma Cell line) were cultivated in Dubelcco's Modified Eagle Medium (DMEM) supplemented with 10% of foetal bovine serum in 75-cm^2^ polystyrene flasks (Costar, Lowell, USA). The cells were incubated at 37°C (95% air/ 5% CO_2_). After three days of cultivation, cells were washed with PBS (Phosphate Buffer Saline) and treated with a trypsin/EDTA cocktail. Trypsinised cells were then centrifuged and collected in fresh medium with serum either for cultivation or future encapsulation.

### Synthesis of living hybrid microcapsules

This section presents two different routes to encapsulate HepG2 within biocompatible capsules or beads under sterile conditions.

#### Method A

Polymeric capsules were prepared by extruding a cell suspension (1.5 10^6^ cells ml^−1^) containing 1.0% of purified sodium alginate into a calcium chloride solution (110 mM). The presence of Ca^2+^ cations act as cross-linkers between carboxylate functions of polysaccharide chains. Since the incubation time of the drops within the calcium chloride solution was quite short (5 min), the gelation process only occured in the outer part of the drops, establishing a solid crust. The capsules were then recovered and 4 of them were put in test tubes filled with culture medium with serum and maintained in CO_2_ incubation in the same conditions than for routine culture.

#### Method B

**Figure 11:**
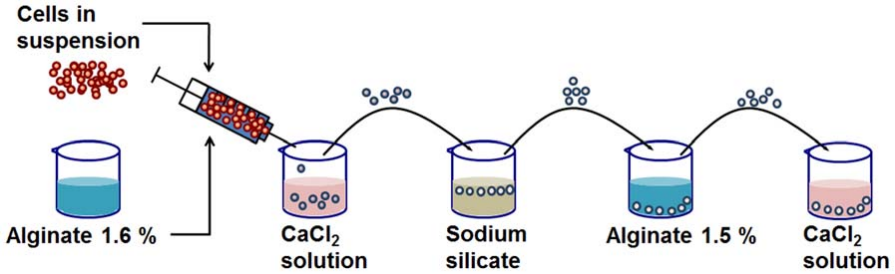
Steps for microencapsulation of animal cells into hybrid alginate-silica beads. Cells in suspension are pre-encapsulated in Ca-alginate capsules and transferred in a diluted sodium silicate solution. The beads are finally coated with a Ca-alginate layer.

Mineralised beads were achieved according to the procedure described in Figure 11. Firstly, cells in their buffered culture medium (pH 7.4) were pre-encapsulated with Ca-alginate (1.6% wt). The final cell density was 1.5 10^6^ cells ml^−1^. The newly formed alginate spheres were added to a diluted sodium silicate solution (1.5 M) for around 30 seconds. A white colour appeared on their surface, and they were collected. Additional external alginate layer was formed by re-suspending the beads in 1.5% sodium alginate solution for 3 min. The outer layer was gelled by the addition CaCl_2_ solution (110 mM).

### Characterization techniques

#### Cytotoxicity test

All cells contain the cytosolic enzyme called LDH (Lactate dehydrogenase). This latter is only located in the cytoplasm. When cells are dying, the plasma membrane becomes permeable and cytosolic components go out (e.g. LDH). A cytotoxicity test can thus be performed by measuring the LDH released in the supernatant. LDH was quantified in samples using the “Cytotoxicity Detection Kit^PLUS^” from Roche Applied Science Laboratory. Briefly, 50,000 cells were seeded in a 24-wells plate. After 24 hours of incubation, potentially toxic chemicals were added to each well. After five hours of incubation, the supernatant of each well was collected. The HepG2 cells, still attached to the bottom of the wells, were then lysed by Triton X-100 (10%) in order to recover the intracellular LDH of intact cells. The extracellular and intracellular LDH activity was then obtained by measuring the absorbance of samples at 490 nm according to the manufacturer's instructions.

### Biocompatibility – Immune test

Monocytes THP-1 were used to investigate the biocompatibility of the capsule. THP-1 cells secrete IL-8 (Interleukin, a primary inflammatory cytokine) in response to proinflammatory stimuli (e.g. stress, viruses, lethal compounds) in their environment. In this case, monocytes THP-1 (commonly used immune defenders for *in vitro* experimentations [49]–[51]) where employed to quantify their proinflammatory response when they are in contact with the components of the hybrid beads. In other words, the amount of IL-8 in the supernatant is an indirect indicator of the biocompatibility of the materials. Briefly, THP-1 cells were cultured in serum-free RPMI medium, for 24 h at 37°C before experimentation to prevent any interference with serum factors. The day after, cells were washed, re-suspended in complete medium and then stimulated with our materials. After 24 h of incubation at 37°C in an atmosphere of 5% CO_2_, the samples were centrifuged at 1000 rpm. for 5 min. The supernatants were collected and stored at −18°C. The IL-8 concentration was assayed by an ELISA test “Quantikine® Human CXCL8/IL-8 Immunoassay” developed by R&D Systems, Inc., according to the manufacturer's protocol.

### Material properties

Beads were characterised by fluorescence microscopy in order to investigate the efficiency of the silicification process. Mineralised beads and alginate capsules were washed with the culture medium and then mixed with 20 µl of 2-(4-pyridyl)-5-((4-(2-dimethylaminocarbamoyl)methoxy)-phenyl)oxazole (PDMPO, 10 µM). After 5 min. of incubation, beads were observed with a fluorescent Multizoom AZ100 microscope system (Nikon). Micrographs were taken at 447/60 nm with a colour camera (DSRi1, Nikon) by illuminating the samples with a 377/50 nm excitation light. In the presence of silica, the unique PDMPO-silica interactions induce an intense blue fluorescence of the material [45].

Morphological and textural properties of alginate capsules and hybrid beads were collected using aerogels obtained from the hydrated materials. For that purpose, the capsules and beads were dehydrated with ethanol, dried through a process of critical point drying with liquid carbon dioxide and degassed at 70 milliTorrs overnight at room temperature prior to the measurements. Specific surface area were calculated using the Brunauer-Emett-Teller (B.E.T.) equation in the low pressure region (*P/P_0_* between 0,05 and 0,25) [52]. N_2_ adsorption-desorption experiment was performed at −196°C with a volumetric adsorption analyser, Micromeritics Tristar 3000. Capsule and bead morphology were examined by field emission scanning electron microscopy (FE-SEM) on a JEOL JSM-7500F electron microscope with aerogel sputter-coated with gold. The chemical composition of the samples was characterised by an energy-dispersion X-rays analysis system using an acceleration potential of 15 kV, and a working distance of 8 mm.

Molecular weight cut-off (MWCO) and accessible porosity of the mineralised beads were determined using fluorescent probes with various hydrodynamic diameters: Dextran-FITC and latex beads carboxylate-modified. Their diffusion gives information about the immuno-isolation provided by the mineralised beads. Briefly, 350 beads (corresponding to 3.0 ml) were synthesized and aged during 1 week, then mixed with a phosphate buffered solution (6.0 ml, pH = 7.4) containing either 0.4 mg.l^−1^ of dextran (10, 70 or 250 kDa) labelled by fluorescein isothiocyanate or 200 µl of green fluorescent latex beads carboxylate-modified (2.5% solids). The changes in the concentration of each substance were monitored at room temperature as a function of time. The experiments were performed during several days and the fluorescence intensity of the probes was recorded with a LS45 Luminescence Spectrometer built by Perkin Elmer: Dextran-FITC (Excitation wavelength = 494 nm, emission = 518 nm), Latex beads (Excitation wavelength = 470 nm, emission = 515 nm).

### Metabolic activity and cell viability

Firstly, the metabolic functions were assessed by monitoring the oxygen consumption in a Clark's cell vessel manufactured by HansaTech. Typically, four beads were added to the culture medium (0.8 ml). The respiration activity of entrapped HepG2 cells was measured just after encapsulation and taken as the reference value (100%), and then over the whole experiment for the next samples.

Secondly, the cell viability was confirmed with a vital dye staining (fluorescein diacetate, FDA). Entrapped cells were incubated within the culture medium supplement with FDA (5 µM) at room temperature for 5 minutes. Micrographs were collected at 536/40 nm with a colour camera (DSRi1, Nikon) by illuminating the samples with a 482/35 nm excitation light using a fluorescent microscope (Multizoom AZ100 microscope purchased from Nikon).

Thirdly, albumin protein secretion by entrapped cells was monitored over time. Every three or four days, the supernatant was collected and replaced by fresh culture medium. Thanks to the “Human Albumin ELISA Kit” developed by AssayPro, the concentration of human albumin was measured and standardised to obtain its daily production outside of the beads.
